# Monochromatic Pupillometry in Unilateral Glaucoma Discloses no Adaptive Changes Subserved by the ipRGCs

**DOI:** 10.3389/fneur.2014.00015

**Published:** 2014-02-05

**Authors:** Claus Nissen, Birgit Sander, Dan Milea, Miriam Kolko, Kristina Herbst, Pascale Hamard, Henrik Lund-Andersen

**Affiliations:** ^1^Research Laboratory, Department of Ophthalmology, Glostrup Hospital, University of Copenhagen, Glostrup, Denmark; ^2^Singapore National Eye Centre, Singapore Eye Research Institute, Duke-NUS, Singapore; ^3^Centre Hospitalier National d’Ophtalmologie des XV-XX, Paris, France

**Keywords:** unilateral glaucoma, melanopsin, intrinsically photosensitive retinal ganglion cells, pupillary light reflex

## Abstract

**Purpose:** To detect signs of a possible adaptive mechanism of the intrinsically photosensitive ganglion cells in unilateral glaucoma.

**Method:** Eleven patients with unilateral glaucoma, classified by automated perimetry (glaucoma: mean deviation <0), were studied by monochromatic pupillometry, employing red (660 nm) or blue (470 nm) light, and by optical coherence tomography of the peripapillary retinal nerve fiber layer. The main outcome measure in pupillometry, the area under the curve (AUC), i.e., the product of pupillary contraction amplitude and time, was determined during and after light exposure in glaucomatous and unafflicted fellow eyes and compared to the AUCs of a healthy, age-matched control group.

**Results:** The AUC to stimulation with blue light was significantly reduced in glaucomatous eyes, both during and after stimulus, compared with that of fellow, unafflicted eyes (*p* ≤ 0.014). The AUC to red light stimulation was reduced during (*p* = 0.035), but not after (*p* ≥ 0.072), exposure in glaucomatous eyes. In the unafflicted fellow eyes, the pupillary response to blue light did not differ from that of healthy controls.

**Conclusion:** The pupillary response to blue light was decreased in the glaucomatous eyes of unilateral glaucoma. No difference was detected between the pupillary light response of the unafflicted fellow eyes and that of a healthy, age-matched control group. Thus no sign of an adaptive mechanism was detected, neither in the glaucomatous nor in the unafflicted fellow eyes, and consequently glaucoma appears to differ from non-arteritic anterior ischemic optic neuropathy.

## Introduction

In glaucoma, irrespective of type, axonal damage of the optic nerve plays a cardinal role. Structurally, this damage may be assessed by optical coherence tomography of the optic nerve head, functionally by automated perimetry.

Recently a novel test-modality, monochromatic pupillometry, was introduced (1–3) based on the sensitivity of the pupillary light reflex to light of different wavelengths, in particular on the different responses to red (longwave) and blue (shortwave) light, making it ideally suited to probe the function of the synaptic and the intrinsic function of the melanopsin containing, blue-light sensitive, intrinsically photosensitive retinal ganglion cells, the ipRGCs (4–6). While the synaptic response generated by the outer retina (cones and rods) produces a fast pupillary contraction, the contraction generated by the intrinsic function is slow. By measuring the contraction during a red light stimulus, the function of the synaptic function may be estimated. By measuring the contraction after a blue-light stimulus, the intrinsic function may be estimated. The technique has been employed in studies on glaucoma (7, 8), showing that the ipRGCs are indeed damaged in this disease. Recently a study on another neuropathy, unilateral non-arteritic anterior ischemic optic neuropathy (NAION), showed signs of damage to the ipRGCs in the afflicted eyes, as expected, but also signs of a possible adaptive mechanism, up-regulating the post-illumination response of both the afflicted and the unafflicted eyes to a blue-light stimulus (9). The present study employs a similar design, in order to refute or confirm the presence of a similar mechanism in high tension glaucoma.

## Materials and Methods

### Patients and controls

Patients were recruited in the outpatient department of the hospital and enrolled in the study as they arrived. Their glaucoma diagnoses were established by at least one experienced senior consultant of the glaucoma section. All patients received eyedrops against glaucoma. None had been operated upon, or had received any peroral treatment for glaucoma. None suffered from cataract, other eye diseases or diabetes, and none of the glaucomas were normal or low tension glaucomas.

The inclusion criterion of the study was an established clinical diagnosis of unilateral high tension glaucoma exhibiting a mean deviation (MD) ≥0 in one eye and <0 in the other, as measured by Humphrey Visual Field Analysis. Fifteen patients were included initially, four of whom were excluded, as they did not meet the criterium of (MD) ≥0 in the unafflicted eye, and hence the final sample was 11 patients. When entering the study, the patients were subjected to a standard clinical eye examination, including determination of Best Corrected Visual Acuity (ETDRS). OCT (Cirrus, Humphrey Instruments, CA, USA), automated visual field analysis, SITA standard 30-2 (Humphrey Instruments, Type 750, CA, USA), digital photo of the fundus and the optic nerve head (Visupac FF 450 plus IR, Germany or Retinal Camera TRC-NW7SF, Mark II, Topcon, Japan), and test for color vision (Farnsworth 15D and Ishihara’s test) were also performed. The average peripapillar retinal fiber layer thickness (RNFL) was computed by the OCT software, based on a 512 × 128 scan, centered on the optic nerve.

A healthy control group, consisting of 11 age-matched subjects without any systemic or ophthalmic disease, was recruited by advertisement and included in the study. None of the subjects in this group used medication known to affect the pupillary light reflex. Before entering the study, they were subject to the same investigational procedures as the patients, including a standard clinical investigation with tonometry and fundoscopy (10). The rules of the Helsinki Declaration were adhered to and the study approved by the local ethics committee. Every participant was informed of the procedures performed and his/her written consent obtained. Patients were included all year round (eight during winter, three during summer), controls from April to September.

### Pupillometer

The pupillometer employed has previously been described together with the procedure used (11).

Briefly, the instrument consists of two parts: an input section, which stimulates one eye for a predetermined time period (usually 20 s) with light of a well-defined wavelength and luminance, and an output section, detecting the area of the contralateral pupil before, during, and after light stimulation. Both sections are controlled by a common computer program and thus synchronized. The area of the pupil is monitored with a frequency of 20 Hz and converted into a diameter, assuming a circular pupil. Light intensity (luminance) was 300 cd/m^2^ for red and blue light, corresponding to 10^14,9^ quanta/cm^2^/s (red) and 10^14,8^ quanta/cm^2^/s (blue) and less for the infrared detecting system. All intensities were chosen well below the recommendations of ANSI-2007 and ICNIRP (12). Initial calibration was performed with the RP-655 spectrophotometer (Photo Research, Chatsworth, CA, USA).

### Examination procedures

Pupillometry sessions were performed in a dark room, in which luminance was controlled by the investigator, as previously described (12). All sessions were performed between 9 a.m. and 4 p.m. One eye was exposed to light, as described below, and the pupil of the contralateral eye video filmed. While the patient was seated and the instrument adjusted, ambient light was mesopic for approximately 5 min. Then, prior to examination, the patient was exposed to darkness for 1 min. The examination session was as follows: 10 s of darkness (baseline pupil), 20 s of exposure to red light and 50 s of darkness (post-exposure). After 5–7 min the entire session was repeated, this time with blue light. First examination session was performed with red light, 300 cd/m^2^, second with blue light, 300 cd/m^2^. Subsequently the red and blue sessions were repeated, this time on the other eye.

### Processing and calculation of the output data

#### Pupillometer

The diameter of the video filmed pupil was the principal outcome measure. A baseline pupil diameter (BPD) was calculated as the mean of determinations during the 10 s in the dark preceding light initiation. The pupillary diameter (PD), obtained during light-on and -off, was expressed relative to the BPD: PD/BPD, yielding the normalized PD, NPD. When light was shone into the eye, the PD diminished from BPD to the PD, i.e., (BPD − PD), which, when normalized [(BPD − PD)/BPD] and summed from time = *t*_0_ to time = *t*_1_, was expressed as: $\Sigma_{t_{0}}^{t_{1}}\left(1.0-\mathrm{NPD}\right)\equiv \mathrm{Area under the curve}\;\left(\mathrm{AU}C_{t0-t1}\right).$

The area under the curve (AUC) was calculated for each of three different time-periods: (1) during the 20 s of light-on (AUC_0–20s_), (2) during the first 10 s after light was turned off (AUC_20–30s_) and (3) from 10 to 30 s after light was turned off (AUC_30–50s_). The larger the AUC and smaller the pupil, the healthier the response to light.

#### Statistical procedure

A normal distribution was assumed for pupillary responses (AUC) and the data summarized as mean and standard deviation (SD) for each group and analyzed with *t*-tests between pupillary responses (AUC) in two different conditions: glaucoma vs. non-afflicted (paired) and non-afflicted vs. control (unpaired). Since perimetry data deviated from a normal distribution, non-parametric tests were employed. MD for unafflicted eyes and glaucomatous eyes was analyzed with the signed-rank test and the MD in unafflicted and control eyes with the Wilcoxon test. AUC data were analyzed by a linear model with absolute baseline pupil size, age, and gender as covariates (9). Normalization of data adjusted for differences in baseline pupil size. Calculations were performed using SAS statistical software (SAS version 9.3., SAS Institute Inc., Cary, NC, USA).

## Results

### Subject demographies

Eleven patients suffered from unilateral glaucoma. Their age was 50–74 years. Duration of glaucoma was 1–24 years, mean 8.7. Seven patients suffered from POAG, three from pigment dispersion glaucoma and one from traumatic glaucoma. The healthy control group consisted of 11 age-matched subjects aged 55–66 years (Table 1).

**Table 1:** **Age and gender of patients and controls together with type of glaucomas of the former**.

	Patients			Controls	
	Age	Gender	Type	Age	Gender
	66	m	POAG	58	m
	63	m	TRAU	61	m
	74	m	POAG	63	f
	74	m	POAG	55	m
	71	f	POAG	66	m
	65	f	PIGM	60	m
	71	f	POAG	60	m
	50	m	PIGM	64	f
	57	m	PIGM	61	f
	58	m	POAG	62	m
	69	m	POAG	62	m
Mean	65			62	
*n*	11			11	
Median	66			61	
Wilcoxon	*p* = 0.1				

*PIGM, pigment dispersion glaucoma; POAG, primary open angle glaucoma; TRAUM, traumatic glaucoma; m, male; f, female*.

### Autoperimetry

The defining factor of entry into the study was the size of the MD. Patients with an MD ≥ 0 dB in one eye (the unafflicted one) and <0 dB in the other (the glaucomatous one) were defined as suffering from unilateral glaucoma. MD in the unafflicted eyes ranged from 0.03 to 1.63 dB, median 0.37 dB, and in the glaucomatous eyes from −1.39 to −32.21 dB, median −8.19 dB. This difference was statistically significant (*p* = 0.001, signed-rank test). The difference between the MD of corresponding, unafflicted, fellow eyes and glaucomatous eyes ranged from 3.02 to 32.53 dB. Healthy control eyes were drawn from a sample of previous controls (10). MD ranged from −1.52 to 0.84 dB, median 0.03 dB. The difference between unafflicted eyes and control eyes was non-significant (*p* = 0.9, Wilcoxon).

### Pupillometry

**Figure 1:**
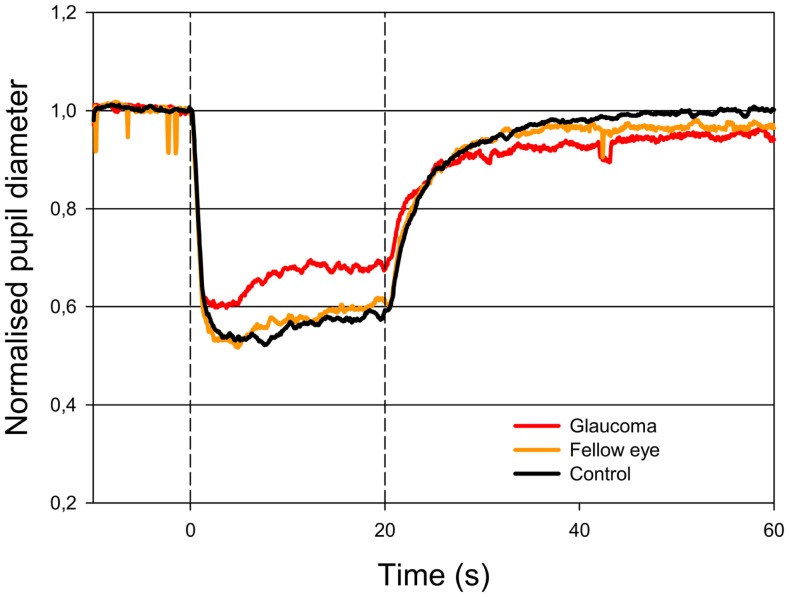
**Pupillary contraction to a red light stimulus (660 nm) as a function of time (s)**. A constant and continuous stimulus of 300 cd/m^2^ was applied at time 0 (first vertical dashed line) and discontinued at the end of the 20th second (second vertical dashed line). The stimulus was applied to one eye and the consensual, pupillary contraction of the other eye recorded. The red graph represents the mean of contractions set off by stimulation in glaucomatous eyes, the yellow, the mean of contractions set off by stimulation in unafflicted fellow eyes and the black the mean of contractions set off by stimulation in control eyes. During light stimulation, contraction is larger in the yellow graph than in the red graph. When the light stimulus is terminated, fairly rapid dilatation ensues and no statistically significant difference is detected (cf. Table 2). Colored graphs represent mean values from 11 subjects suffering from unilateral glaucoma, the black graph the mean from 11 healthy control eyes. The AUC is the area between the horizontal line: NPD = 1.0 and the graph in question.

**Figure 2:**
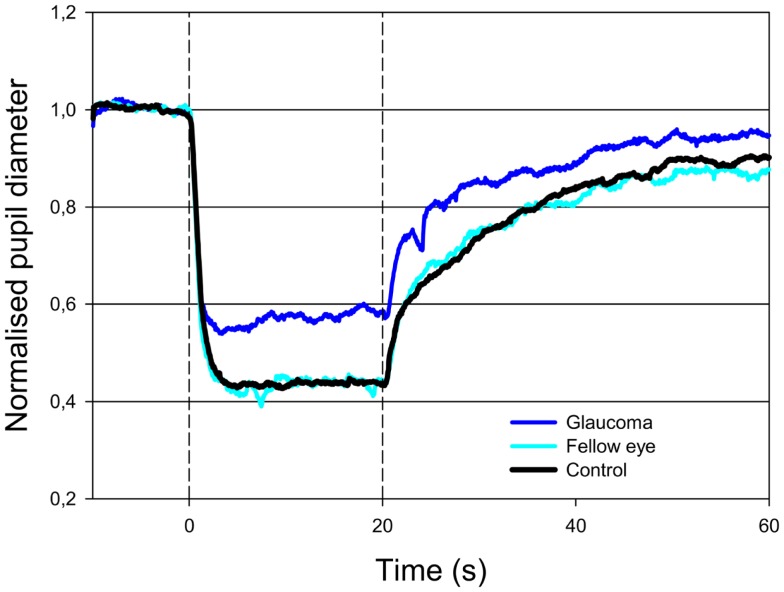
**Pupillary contraction to a blue-light stimulus (470 nm) as a function of time (s)**. Time period, stimulus luminance, and size of input pupil as in Figure 1. The light blue graph represents pupillary contraction set off by stimulation of the unafflicted fellow eyes, the dark blue graph that set off by stimulation of the glaucomatous eyes. In comparison with the graphs in Figure 1, contraction is larger during light-on in both graphs, and post light stimulus redilatation is far slower than that due to red light. Proportionally, the difference in contraction between the glaucomatous eyes (dark blue graph) and the unafflicted eyes (light blue graph) during blue light-on is equal to that detected in the red graphs (cf. Table 2). After light termination, the difference between the two blue graphs is slightly smaller than during light-on. Results represent mean values from 11 subjects suffering from unilateral glaucoma and the black graph the mean from 11 healthy control eyes.

The AUC to light stimulation (AUC_0–20s_) in glaucomatous eyes was reduced to both red and blue light as compared with unafflicted fellow eyes (Figures 1 and 2). The AUC to blue light was reduced from 10.80 (unafflicted) to 8.29 (glaucomatous) and to red light from 8.86 to 6.64. These reductions were significant (*p* = 0.014, blue and *p* = 0.035, red; *t*-test, Table 2). Both post-illuminatory AUCs (AUC_20–30s_ and AUC_30–50s_) to blue light were significantly reduced in glaucomatous as compared with unafflicted fellow eyes (Figure 2; Table 2). After termination of exposure to red light, redilatation was rapid, and no significant difference was found between unafflicted and glaucomatous eyes (AUC_20–30s_). The AUC_30–50s_ for glaucomatous eyes were slightly larger than that for unafflicted eyes, but the difference did not reach statistical significance (Figure 1; Table 2). The AUCs of the unafflicted fellow eyes were not different from the AUCs of age matched, healthy, control eyes, irrespective of the color of the illuminating light (Table 2), apart from the late response, AUC_30–50s_, to red light, where a significant difference was found.

**Table 2:** **The pupillary responses during and after light stimulation in 11 patients (unafflicted and glaucomatous eye) and in 11 healthy, age-matched controls**.

AUC									
	0–20 s light-on			20–30 s light off			30–50 s light off		
	Blue	Red	Blue–red	Blue	Red	Blue–red	Blue	Red	Blue–red
**UNAFFLICTED**									
Mean	10.795	8.848	1.947	3.496	1.776	1.720	3.927	1.186	2.741
SD	1.182	1.886	2.214	0.868	0.513	1.033	2.027	0.552	1.986
**GLAUCOMATOUS**									
Mean	8.287	6.636	1.651	2.330	1.491	0.839	2.364	1.848	0.515
SD	2.833	3.011	1.103	1.101	0.760	0.721	1.522	1.353	1.554
**CONTROL**									
Mean	10.495	8.447	2.048	3.461	1.749	1.712	3.445	0.637	2.808
SD	0.928	1.732	1.214	0.669	0.602	0.800	1.830	0.386	1.747
***t*-TEST UNAFFLICTED VS. GLAUCOMATOUS (PAIRED)**									
*p* =	0.014	0.035	0.699	<0.001	0.321	0.013	0.001	0.072	0.004
***t*-TEST UNAFFLICTED VS. CONTROL (UNPAIRED)**									
*p* =	0.516	0.609	0.896	0.917	0.913	0.983	0.565	0.015	0.934

*The pupillary contraction is summarized as the mean and SD of the area under the curve (AUC) for three intervals: 0–20 s during light-on, 20–30 s, after light is turned off and 30–50 s, after the light is turned off. Significant differences were found between unafflicted and glaucomatous eyes during light exposure to red and blue light and after termination of blue light. For the comparison of unafflicted eyes to healthy controls, the differences were non-significant, apart from a significant difference at 30–50 s, as red light was turned off. AUC (blue–red) is included for comparison*.

The baseline pupils in unafflicted eyes and glaucomatous eyes were not significantly different (unafflicted, mean 28.45 mm^2^, SD 4.9; glaucomatous, mean 30.73, SD 6.63; *p* = 0.097; *t*-test). In healthy control eyes, the mean pupil size was 38.52 mm^2^, SD 7.55, which was significantly larger than that of unafflicted eyes (*p* = 0.0014). When the AUCs for healthy control eyes and non-afflicted eyes were analyzed in a general linear model, however, the baseline pupil as well as age and gender were all non-significant covariates (*p* > 0.1, data not shown).

### OCT

The average thickness of the RNFL was 81.73 μm (SD 14.28) in unafflicted eyes and 60.18 μm (SD 8.54) in the corresponding glaucomatous eyes, or a 25% reduction. This difference was statistically significant (*p* = 0.001, *t*-test).

The average thickness of the RNFL in control eyes was 86.91 μm (SD 8.01), which was not significantly different from that of the unafflicted eyes (*p* = 0.31, *t*-test).

## Discussion

The aim of the present study was to compare the function of the ipRGCs in glaucoma to that already published in NAION, employing the same method: monochromatic pupillometry, performed on the same pupillometer.

The basic design of the study consisted in comparing the pupillary responses to red or blue light in the glaucomatous eyes with those of the unafflicted fellow eyes and further to compare the responses of these unafflicted eyes with those of an age-matched healthy control group. If adaptation – as defined in the NAION study – was present, then the responses of the glaucomatous eyes should equal those of the healthy control eyes, while the responses of the unafflicted fellow eyes should exceed the responses of these control eyes. The main output response of the present study was the AUC to red light and to blue light. AUC was chosen, because it sums all pupillary information and therefore by its very nature is robust to deviations such as blinks or pupillary oscillations.

In the present study, the function of the melanopsin containing ganglion cells, the ipRGCs, was examined in strictly unilateral glaucoma. We observed a decrease in the PD both during light stimulation (light-on phase) and after light termination (light-off phase), when the stimulant light was blue, but only during light-on, when the light was red.

This suggests that the intrinsic function of the ipRGCs is damaged in glaucoma, and, since the pupillary response during red light stimulation is taken as a measure of synaptic function, that this function is equally damaged in glaucoma. The present study shows a significant reduction of 25–30% of pupillary response (AUC) during red or blue-light exposure and 33–40% after light exposure to a blue light in the glaucomatous eyes, indicating a loss of synaptic and melanopsin driven response in the glaucomatous eyes. A significant reduction of blue post-illuminatory responses is in fair agreement with that presented by Kankipati et al. (7). Feigl et al. (8), on the other hand, present a smaller change in glaucomatous eyes, but their outcome measures are not strictly comparable to those of the present study, as their design did not admit of a direct comparison of blue light in a control eye, using instead the response to red light as control. By examining unilateral glaucoma, a direct comparison of responses to both blue and red light was possible, thus avoiding the influence of inter-individual variation and circumventing the use of red light as a substitute for blue cone stimulation. We also calculated the blue–red difference using the present data. Essentially the same conclusions may here be drawn as with the unilateral approach, apart from the AUCs_0–20 s_, blue and red light, where the unilateral approach produces a significant difference between glaucomatous and unafflicted eyes, while the difference approach does not. In conjunction with the decrease in pupillary response, a thinning of the peripapillar retinal nerve fiber layer thickness was observed.

When the AUCs of the unafflicted eyes were compared to those of control eyes, no significant differences were found between the two groups. This was in contrast with the findings in a similarly designed study on (unilateral) NAION. In that study the post-illumination AUCs of the NAION eyes to blue light (indicators of ipRGC function) did not differ from those of the healthy control eyes, while the AUCs of the unafflicted fellow eyes were greater than those of controls. Similarly, the AUCs in unafflicted eyes were up-regulated during red light exposure (indicators of synaptic function). In other words, both NAION eyes and unafflicted fellow eyes were up-regulated (9). In the present study, no sign of such a mechanism was in evidence, even though the study design, equipment employed, sample size, age-groups, output measures, and possible biases were similar.

A limitation of the present study was the small number of subjects and the fact that the healthy controls were examined approximately 1 year earlier than the unafflicted eyes. The paired design, however, reduced variation in the comparison between glaucomatous and unafflicted eyes. The small number of subjects in the patient and control samples is the most plausible reason for the significant difference between the AUCs (red light off, 30–50 s) of unafflicted and control eyes (Table 2). Differences between sampling seasons of patients (three sampled during summer, eight during winter) and controls (sampled from April to August) might bias the results. No patients suffered from significant age-related lens changes [cf. (10)].

The observation and the concept of adaptation are new. Explanations must necessarily remain speculative at present. Provided, however, that adaptation can be confirmed, the only evident difference between the NAION study and the actual study on glaucoma is etiological: NAION is due to a deficit in vascular capacity, high tension glaucoma probably first and foremost to a rise in intraocular pressure. Since the retinal nerve fiber damage – judged from the glaucoma results – not *per se* gives rise to adaptation, the up-regulation might be initiated elsewhere: could it be vascular? This interesting question must await further elucidation.

In conclusion, the present study on unilateral glaucoma confirms previous findings of a decreased post-illumination response to blue-light exposure in glaucoma. The study, however, also reveals a similarly decreased pupillary response during exposure to red light, and a reduction in the peripapillary retinal nerve fiber layer, strongly indicating that ipRGC function, both synaptic and intrinsic, as well as retinal ganglion cell function in general are damaged in glaucoma. Being a study of unilateral glaucoma in a sample of aged individuals with a mean observation time at detection of 9 years, it is finally concluded that no sign of damage to the contralateral, unafflicted fellow eye was detectable and that unilateral glaucoma therefore appears to be a genuinely unilateral disease, which, contrary to the results of a recent pupillometric study on NAION, shows no sign of any adaptive mechanism, up-regulating the pupillary light reflex.

## Conflict of Interest Statement

The authors declare that the research was conducted in the absence of any commercial or financial relationships that could be construed as a potential conflict of interest.
